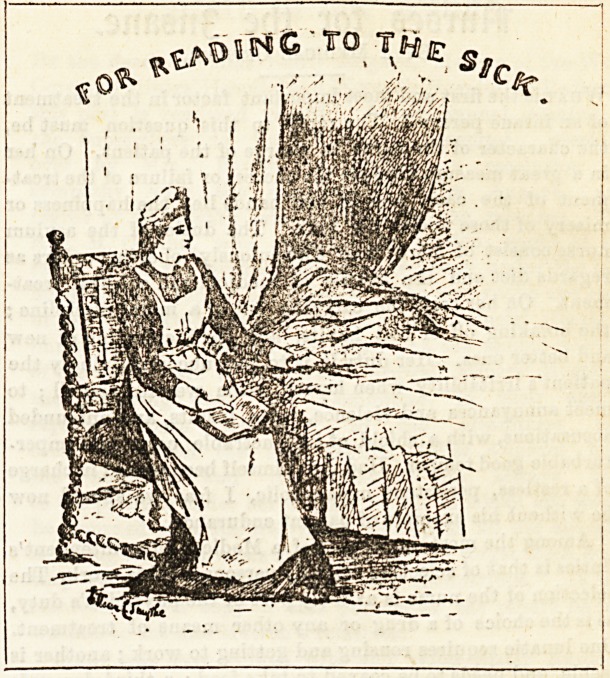# The Hospital Nursing Supplement

**Published:** 1892-03-19

**Authors:** 


*The Hospital*, March 19, 1892. *Extra Supplement.*

### "The Hospital" Nursing Mirror.

Being the Extra Nursing Supplement of "The Hospital" Newspaper.

Contributions for this Supplement should be addressed to the Editor, The Hospital, 140, Strand, London, W.C., and should have the word "Nursing" plainly written in left-hand top corner of the envelope.

### En Passant.

NURSES' CASES.—We happened to visit Allen and Hanburys', in Vere Street, last week, and were much struck by their cases of instruments for nurses. Two especially seemed worthy of note; the first a square case for hanging at the belt, very completely fitted, and with a flap so that it could be shut up when out of doors, or while dusting, and so the instruments kept clean. The second was a pocket case, suitable for private nurses, whose patients are apt to be overawed by the appearance of instruments worn from the belt. Both cases were very neat and strong and complete.

ST. PATRICK'S HOME.—The Archbishop of Dublin presided at the annual meeting of this Home. The report and statement of accounts for the past year were submitted to the meeting, and showed that the income of the institution had been £1,103 15s. 2d., and the expenses had been £827 11s. 3d. The Home has again received two generous contributions in consequence of the affiliation with Queen Victoria's Jubilee Institute, £100 each from Mr. Rathbone and Lord Iveagh. The Chairman in his speech gave some particulars of the work done by the nurses. He said the Home now had under its roof eight nurses, including the Superintendent. Of these, three belonged to the institution and five were probationers, and there was a prospect of another joining their number. The probationers were not to be looked on as useless or helpless members of the staff of nurses, as they had all been thoroughly trained in hospital work before coming to the institution. Last year these nurses had attended over 500 separate cases, and each month they have paid over 1,000 visits to the sick poor.

A RESIDENTAL CLUB.—Of nurses it certainly cannot be said, as it is of other women, that they lack the power to combine. They have combined to excellent purpose in the Co-operation they have for years maintained a charming little social club in Buckinghamshire Street, Strand; and they are now going to start a residential club at 92, Charlotte Street, Fitzroy Square. It is an open secret that one of the main supports of this new club is an active member of the Co-operation Committee, and a well-known holder of the L. O. S. diploma. Other nurses are helping the scheme, and at least two Matrons, so we predict success for it; though we confess that the tariff appears to us so low that we cannot see how it can pay. We quote from the prospectus. "Nurses' Residential Club: established to meet the requirements of nurses living in single rooms who desire a place where they can obtain meals, and meet their friends. Also for nurses who require an occasional bed and a permanent address. Entrance fee; 10s. 6d.; annual subscription, ordinary members, 10s. 6d.; members wishing to use the club as a permanent address, and to have letters and parcels forwarded and enquiries answered, £1 1s.; two satisfactory references must be given by each member. The Committee retain the right to refuse membership, should such refusal appear to them necessary for the well-being of the club. Club members belonging to the Nurses' Co-operation can obtain bed and storage for boxes on the 25th March. The club will be formally opened early in April." Evidently those Co-operation nurses are to have the best of it as usual! We shall not quote the tariff, or the Hon. Secretary will have such a rush of applications she will never be able to answer them; but as a specimen take this—"Tea—with jam or cake 6d."

BOLTON INFIRMARY.—On March 10th the annual meeting of friends of this institution was held, when it was reported that the number of cases treated during the year had been 954 in-patients, and 3,104 out-patients. There is a deficiency of £900 on the year's working. The Committee record their satisfaction with the labours of Miss Anstey, the Matron. Mr. Cooper referred to the fact that three of the nurses—Sarah Gregory Finch, Maud Evelyn Slater, and Ellen Rose Holliday—had taken during the last week the First Class Certificate and maximum gratuity for their three years' training. The examinations passed bespoke the highest efficiency. He was sorry they had no opening for these nurses, but hoped to see them back at some future time.

SHORT ITEMS.—Miss Kate Whistler, sister of the well-known artist, and for many years a member of the Naval Nursing Force, is leaving her post at Plymouth in order to be married.—We learn with pleasure that a thoroughly good Matron has been appointed at the Eastern Hospital, and hope she will get a good staff of nurses to support her.—Miss Ward (Sister Edith) has gone abroad with the Prince and Princess of Wales; Sister Edith helped to nurse Prince George and the late Duke of Clarence; she was at one time connected with the Hollond Institution at Nice. —No district nurse has yet been found for the Island of Gigha; are Scotch women no longer capable of self-sacrifice? —On the 10th instant the Ryde District Nursing Association affiliated with the Jubilee Institute. The Queen has sent the association a donation of £5.

NURSING GUILDS.—The seventh report of the Richmond St. John Nursing Guild is just out; the members are ladies who have passed the St. John Ambulance nursing course, and who are willing to practice the theories there taught on their poor neighbours. We are glad to see that one of the members has thus learnt her ignorance and has gone to the Middlesex as a probationer. Nursing guilds or corps on parallel lines are now working in Guernsey, at Oldham, Newark, South Shields, and Ramsgate. And during this year the Richmond rules and papers have been sent by request to Padiham, Barnsley, West Hartlepool, and Farnham; and Sir Vincent Barrington wrote for copies for a meeting at Portsmouth. Whether this may result in nursing guilds being started in either of these places is not yet certain. In Richmond the guild is better than nothing at all, as that luckless town possesses no district nurse.

DISTRICT NURSING.—District nurses are certainly the prime favourites just now. There is a long discussion on their merits going on in the *Charity Organisation Review*, which is, on the whole, condescendingly favourable. At Forfar, and at Fort William, meetings have been held in favour of establishing Queen's nurses in the towns. Miss Guthrie Wright, the energetic Secretary of the Scottish branch of the Jubilee Institute, attended the Forfar meeting, and made a long and able speech. In concluding, Miss Wright said that amongst the benefits of having such an institution were that they had a guarantee of having a high standard of training, and of having nurses with an aptitude for district work. The nurses first came for a month's probation; and if they were found unsuitable, unsympathetic, indiscreet, or gossipy women, they were not retained, and if, on the other hand, they did not like the work, they would leave. The nurses had also the satisfaction of working in a national institute in which they had the benefit of being trained, and, although it was a national ramification, they were not dependent on one another; and, lastly, it was a great satisfaction to be working in an institute inaugurated by Her Majesty—working with and under her, and carrying out her wishes in the matter.

### On the Nursing of Children.

VI.—SPLINTS (*continued*).

To wash a child in a splint requires a good deal of intelligence, and it is not always as thoroughly done as could be wished. Of course, no one desires any risks to be run in the handling of recent fractures, but a great many surgical cases can be washed without any risk at all.

If the skin be bathed and properly dried under the body band, the child is made much more comfortable, and the same thing applies to the axilla and soles of the feet.

Untrained and half-trained people take often credit to themselves for some fancied personal superiority, when they say they are "so afraid of hurting the dear child," that they have not attempted "to do much to him," and they think that the thoroughly-trained nurse is devoid of this charming sensitiveness, because she can conduct the necessary ablutions calmly and quietly, with no shirking of those folds and creases, which are always dirt-traps.

The thorough and careful washing which steady, experienced hands perform, gives comfort, not annoyance, to the fidgetty little patient, who soon learns to appreciate the relief of it, and to offer no opposition, although he may still shrink a little when the damaged limb is approached.

It is very hard for anyone to have to lie all day and all night fastened to a splint, but it is far worse for children than for adults, for it is natural to the former to be always moving. Therefore, great pains should be taken to remove every little additional trial, and every unnecessary discomfort.

If a child has complained of anything hurting him in any spot covered by a bandage, it should always be mentioned at the surgeon's next visit; even if the complaining has ceased there may be mischief going on, and a sore is easier made than healed.

When a plaster-of-Paris, gum and chalk, or similar splint succeeds the first one, fresh anxieties arise, and the child who has been treated in a hospital should never return home without his parents being forcibly warned to attend to the earliest complaint of discomfort, and to watch the skin round the edges of the splint for any redness or abrasion, and the foot for any sign of swelling, and if such there be, to bring the child to see the doctor at once.

Few things are more annoying than to see a child who was sent home clean and sound, brought back, not only very dirty, but with some sore which his mother calmly announces has "been coming larger every day for this last week or more."

When "Sayre's jackets" are removed there is often a sad state of skin revealed, for however impressive and frequent the warnings given to the friends to take heed to the first signs of discomfort, it seems impossible to get the importance of this grasped by the poor folks whose procrastination is as deep-rooted as their ignorance.

When the "jacket" is to be put on great care should be taken to have a thoroughly good vest ready for the surgeon's bandages to cover, and it is a nurse's duty to see that the one supplied is a perfectly satisfactory fit, or, better still, she should have two at hand, as some surgeons will use both. By this plan it is possible occasionally to change the one next the skin, as a clean one can be stitched firmly to its edge, and thus can be gently and slowly dragged into position when the soiled one can be unstitched and removed.

It is often necessary to fasten a child down in bed to further the union of bones, or, as in hip disease, to enable the extension to work properly, and for many other reasons, too; but in all cases the greatest care should be taken to make the arrangement as little irksome as possible; of course, it must be always painless. The bands which encircle the arms should be large enough for comfort, and small enough to keep their proper position, and the flat leathern strap which keeps these in place, and which is perhaps the best thing for the purpose, should be wide and perfectly smooth, and fastened tightly over the mattress. The ends of the leather should be secured under the cot, where it is impossible for the patient to reach them, otherwise the temptation becomes irresistible, and but few knots can puzzle the active littttle fingers which can devote unlimited time to loosening them. The draw-sheets should be carefully chosen, and frequently examined for wrinkles; a patch or a hard seam is quite sufficient to distress the tender little back, and even to do considerable harm if left in contact with it for some hours. In fact we may well end by saying that from the first moment of its adjustment to the last day of wearing a splint both it and the child will require careful supervision and plenty of our patience.

### Medical Opinion on Nurse Registration.

In an article on this subject the *Glasgow Medical Journal* gives the following:—

Our readers may remember that the application of the Royal British Nurses Association to the Board of Trade for registration without the word "limited" was refused. We have already strongly expressed our opinion that it is not desirable for nurses to be registered in the manner aimed at by this Association, and we have no doubt that the reasons which caused the President of the Board of Trade to refuse the licence will lead to a similar result on the part of the Privy Council. We know that the Board of Trade has been known to alter its first decision upon a re-consideration of the case, but the opposition to the British Nurses' registration scheme seems to have been so powerful and general, that it has not been possible to approach this department of the Government again.

The object of the Association, says a leader in the *Daily Telegraph*, "is the elevation of the nursing profession by some recognised method of registering the fit and eliminating the unfit." We hold that registration will never effect this. If "eliminating the unfit" means the maintenance of a dead level of uniformity at the lowest level possible, then perhaps registration may effect something, but how this can be regarded as in the slightest degree tending towards the "elevation of the nursing profession" we entirely fail to see. We do not think the reference in the same article to the registration of "the plumber who mends our drains" is particularly happy in the present connection. We could understand it if the highest ideal of sick-nursing were to regard it as a mere trade. But nursing is not, and can never be, a trade; and the moment nurses begin to form a trades' union a great measure of their usefulness and influence is lost. The institution of a register would be no guarantee that only fit women would obtain the necessary certificate We have not at present any basis of union on which a system of registration could be built up, even if registration were desirable, which it is not. It is the individual woman and not the register-ticket that is the important thing for physicians and surgeons and the public to consider. Besides all this, the granting of such a charter would place the absolute control of nursing in these islands in the hands of a self-constituted private company—a control to which it has really no claim whatever.

### Wants and Workers.

*Spinal Carriage.*—We want very badly a spinal carriage for the children with hip disease to go out in. Perhaps some one has an old one to spare, or perhaps some one will give us the money to buy one?—*Matron, Children's Convalescent Home, Clifton Road, Weston-Super-Mare.*

### Nursing Uniforms.

### III.—RUSSIAN NURSES.

The above two figures represent a Russian Red Cross nurse and a Russian fever nurse.

Save in the case of the Red Cross Society nursing in Russia is not far advanced, but the members of the Red Cross are trained theoretically and practically, and work in the chief hospitals, including all the military ones. They are mostly women of the lower classes, though, during the Russo-Turkish War, a large number of ladies joined their ranks for temporary service. The uniform is very pretty; the cap is a modified form of that worn by the Sisters of St. Vincent de Paul, and the red cross is worn not only on the left arm, but on the breast of the apron. The dress is either grey or black, and it is unusual for Russian nurses to wear washing frocks. In the military hospitals these Sisters fulfil much the same duties as our army Sisters, and have under them male nurses, or orderlies, to do the rough work.

Our readers have probably all seen Mr. Kerman's book on "Siberia," in which he describes the horrors of the prison hospitals on the route followed by the exiles. There are no nurses, no nursing of any sort whatever, and the death-rate is something terrible. On this and on other similar subjects we shall probably have more information soon from Miss Kate Marsden, who is travelling in Russia, and inspecting the hospitals and leper houses.

The Russian fever nurses have a strange method of avoiding infection. They wear an overall and hood, and across the mouth a piece of carbolised gauze. Whether they do this in hospital or not we cannot say; the above picture is of a visiting nurse, or sort of district nurse for the higher classes. It is certainly not our method of avoiding infection, and such a startling dress would be likely to alarm friends and patients alike.

On the whole, a glimpse at foreign uniforms only serves to increase our comfortable British pride in our own superiority.

*C. R. C.*—We are afraid we cannot help you to the employment you want, but we have noted your address.

*Examination Questions.*—The awards will be made next week. The delay is caused by the necessity of going over back papers to see who has won the prize for industry.

*Book About Drugs.*—The most common drugs are described in "The Nurse's Dictionary," price 2s., from this office.

*E. M. M.*—"Tokology" can be had from the office of *The Woman's Herald*, 86, Strand, W.C.

*A Medical Nurse.*—See "The Hospital Annual" and our back numbers. There are not many American hospitals which now engage English nurses. You can join any of the directories; there are no institutions in America.

### "WINGS."

The word "wings" suggests to us the easy, graceful movements of a bird passing through the air without effort, and as we picture its happiness and freedom, we also long to be soaring in the bright blue sky, leaving the earth and its troubles far behind.

We are not singular in our wish, for the Psalmist exclaims in his trouble, "Oh! that I had wings like a dove, for then would I flee away and be at rest," and as that gentle bird ever seeks quiet in its own home, so would he hasten to escape from the stormy wind and tempest which beset him. And our souls may find "wings" with which to fly away from our troubles, though our bodies be bound down by affliction to our beds. The wings of prayer will carry us out of ourselves, and land our petitions for mercy and help safe at the footstool of the Almighty. It may seem as difficult to some of us to pray as to skim through the blue arch of heaven; but let us consider whether the bird we so envy could always take such a bold and lengthened flight as we are picturing. It was once a weak and timid nestling, fed from its parents' beak, and when it had grown stronger required teaching how to use its pinions, and to be encouraged to throw away its fears and launch into space by itself.

We have a loving Father who does the same by us. He feeds our souls though we do no realise it; He puts into our hearts good wishes, which teach us to ask all things from Him, and when trembling and cast down, we essay a few petitions, He encourages us by answering them beyond our hopes, till we grow strong and can launch forth into the wilderness of life, upon the wings of the morning, feeling sure that wherever we go, or whatever may befall us, God's hand will lead us, and His right hand will hold us.

How independent we are of our poor feeble bodies, when we can soar away in prayer to the Throne of Grace! What matters it to us if they lie helpless on the sick couch, if our souls are basking in the rays of the Sun of Righteousness, and when we again settle upon earth everything seems changed to us; our very faces are transfigured, and those about us note that we have been with Jesus. From making our wants known to God in prayer we shall go on to praising Him for the mercy both to our souls and bodies, which endureth for ever.

We will copy the lark, which "sings as it soars, and soars as it sings."

"Type of the wise who soar but never roam."

So will our instincts be

"True to the kindred points of Heaven and home."

### Nurses for the Insane.

By a Medical Officer.

What is the first and most important factor in the treatment of an insane person? The reply to this question must be, the character of the nurse in charge of the patient. On her in a great measure depends the success or failure of the treatment of the case, and in her hands lies the happiness or misery of those under her care. The duties of the asylum nurse consist of more than scrupulously obeying orders as regards diet and the details of medical and surgical treatment. On her rests the carrying out of a moral discipline; the breaking off of bad habits, and the inculcating of new and better ones. Her duty it is to bear uncomplainingly the patient's irritability when herself often wearied and ill; to meet annoyances and violence, open insults, and unfounded accusations, with a shield of impenetrable calm and imperturbable good temper. Had Job himself been placed in charge of a restless, persistent melancholic, I fear we should now be without his example of patient endurance.

Among the most important of a Medical Superintendent's duties is that of selecting suitable persons for this work. The selection of the nurse is as truly part of the physician's duty, as is the choice of a drug or any other means of treatment. One lunatic requires rousing and getting to work; another is feeble, and needs to be coaxed to take food; a third demands strict but kindly discipline. Yet we have heard it urged that this part of a Superintendent's duties should be taken from him, and that he should not have his time wasted in selecting or controlling his most important agencies for cure.

How is it that this work has not enlisted volunteers from the class of educated women, who are thronging all other avenues open to them? Ought the unpleasant nature of many of the duties of a nurse to the insane to be a sufficient reason why women, educated and refined, should not venture into that, for them, practically untried field? How requisite are all the qualities they might bring to the work; tact, refinement of feeling, sympathy, &c. How much anxiety would be saved to medical men, and how much more efficacious their efforts if seconded by a staff of women, capable of making accurate observations, and of forming a trustworthy judgment. Ask any Medical Superintendent which post he finds it most difficult to fill satisfactorily, and the reply will be: "The higher female ones." True women, gifted with clear heads and sympathetic hearts, are wanted. A practical knowledge of a nurse's duties, her many trials and difficulties, is requisite. One who can be amongst her staff, directing and aiding, and yet not exactly of them, is what is needed, and is what is not easily obtained. Many women, excellent in many respects, can be found among the present workers in our female wards, but want of education and inability to command respect and obedience unfit them for the more responsible posts. In entering asylum work, the most difficult lesson for the higher class woman to learn would be that of unquestioning obedience and subordination to those above her. The attention that is paid to apparently trivial matters which, she will imagine, should be left to her own discretion, will be likely at first to hurt her *amour propre* and ruffle her feelings. She would find much to do which to her was repulsive, and she would soon understand that real hard work lay before her. Many things also would be seen and heard well calculated to shock and turn back any faint-hearted waverer.

An asylum is a hospital for the recoverable, combined with a home for those unfortunates who, from the incurable nature of their malady, are doomed to live apart from their fellow-creatures, and who yet require constant care and supervision. This home is to be made as cheerful and as little like a place of enforced retirement as possible. Every means is tried to restore the afflicted before they reach a stage when no hope of restoring them to life outside can be entertained. Very erroneous ideas of the interior of an asylum of the present day still persist. The old repressive treatment has gone, and enlightened methods rule. The cruelties of a semi-barbaric past are nearly forgotten. So much has the treatment of lunacy turned its back upon itself that calmer minds now reflect whether in casting away the errors and abuses of our forefathers, we have not also lost the germ of truth that lay beneath them. It may be that the adoption of manual and chemical restraints in place of the older methods have been the means of inflicting most exquisite suffering upon many. The end attained undoubtedly in many cases might have been gained in a more truly humane fashion by a judicious employment of means now tabooed because unfashionable and contrary to the sentiment of the age.

Much has been done in the way of social life among the insane, and much more might still be done. This, the work of providing amusement and recreation, is, paradoxical as it may seem, in reality the most wearisome and trying of all asylum duties. Enthusiasm gets killed in the frequent meeting of similar faces; in the constant effort to amuse and interest, and at the same time to appear amused and interested. Much leisure time must be given up, and, hardest of all, it must be done cheerfully and with a good grace, or all is useless. The least relaxing of interest shown by those at the head is quickly reflected through nurses down to the patients, and instead of the evening's entertainment being brisk and cheerful it becomes flat, stale, and utterly unprofitable. When these gatherings do not take place we find the social side of the lunatic's life dies from inanition. Dementia appears before its time. The patient gets day by day deeper in the dull rut of routine: works, eats, and sleeps like an automaton, and is indifferent to everything. Perhaps from the philosophic point of view his is the gain inasmuch as he has reached true happiness in that he has become oblivious to all the manifold worries of daily life.

Might not women be employed in male wards, and especially in those for the sick? This has been tried but has not been found to be a success. There are some male patients who are quite unfit to be left in charge of women, and thus the additional expense of two sick wards would be incurred. Probably most success has attended the plan of having small wards of selected patients in charge of married couple. But even here difficulties with regard to leave of absence, &c., are encountered.

There ought, in our large asylums, to be openings for female dispensers and for the trained masseuse.

(*To be continued.*)

### Where to Go.

A course of five lectures on "Domestic Hygiene" will be given at the Parkes Museum, 74a, Margaret Street, W. (opposite All Saints' Church), four by A. T. Schofield, M.D., M.R.C.S., on the following days, and one by Rev. J. R. Byrne: Tuesday, March 22nd, "The Phenomena of Life"; Friday, March 25th, "Food and Dietetics"; Tuesday, March 29th, "Physical and Mental Training of Children"; Friday, April 1st, "The Effects of Posture on the Health of School Children"; Tuesday, April 5th, "The Hygiene of District Visiting." The lectures will commence at 3 p.m., and will be illustrated by diagrams, microscopic specimens, food collections and school desks and fittings, and an exhibition of physical drill will probably be arranged for. Afternoon tea will be served after each lecture, when any questions concerning the lecture may be asked. A written examination will be held at the close of the course, and certificates granted to successful candidates. Particulars of the examination is given on the fourth page. Her Royal Highness the Duchess of Albany, patroness of the institute, has graciously expressed her intention of being present at some of the lectures, and has consented to present the certificates at a special meeting to be held in June or July. Fee for the course, including examination, 10s.; family ticket, to admit three, 25s.; single admission, 2s 6d.

### Everybody's Opinion.

### DISTRICT NURSING DISAGREEMENTS.

Miss Lutticнau writes:—May I say, in answer to Mr. Percy Wigram, that I had no intention of deprecating the importance of the East London Nursing Society; but do not think it has ever been considered a branch of the Metropolitan and National Nursing Association. I cannot find it so called in any of the reports of that society, nor is it mentioned in any published lists of branch homes. The East London Nursing Society was founded in 1868; the Metropolitan and National Nursing Association in July, 1875. Can a branch grow before its parent stem? In 1875 the East London Nursing Society had seven nurses at work, and the first report of the Metropolitan and National Nursing Association mentions the fact that it had incorporated with itself the East London Nursing Society, although, in the same paragraph, attention is drawn to the important particulars in the system of the latter wherein the conduct of its work differs from the lines adopted by the Metropolitan and National Nursing Association. It is this difference which has led us to consider ourselves as doing the "largest work of the kind in London." We have not thought of comparing ourselves with the East London Nursing Society, differing as it does from the North London Nursing Association in origin and system. Of the homes founded under the auspices of the Metropolitan and National Nursing Association, the North London Nursing Association, formerly called "The Northern Branch," does the largest work. I am sorry that I should have appeared in any way to desire to underrate the work of the East London Nursing Society, which was not in my mind when I wrote. Further, Mr. Percy Wigram says that in 1881 the East London Nursing Society, like the North London Nursing Association, resumed a separate existence; true, indeed, for the older institution, but not for us, since one cannot resume what one has not before possessed. In February, 1881, the North London Nursing Association was cut off from the Metropolitan and National Nursing Association as—so it then seemed—a worthless branch, though, owing to its many friends in the north of London, it has since greatly increased its sphere of usefulness.

### To a Nurse.

With faithful heart go forth and ply thy calling
From day to day.
The Master's love is round thee, He will keep thee,
And be thy stay.

And if He bid thee walk in shady places,

Have thou no fears.

The "Man of Sorrows," doth He not remember

This vale of tears?

Doth He not stand beside each sufferer's pillow,
And say to thee—
"All that thou doest, child, for these My brethren,
Ye do to Me?"

O, if thy heart shrink back as all unworthy

So high a task,

Remember His own word—"A cup of water

Is all I ask."

Go, with thy smile, then, many a sad soul lighten;
Speak soft and low.
A gentle word may heal a heart that's broken,
Give joy for woe.

God does not bid us look at life's long sorrow,

But each day take

And do its duties faithfully and sweetly,

For His dear sake.

M. Day.

### Death in our Ranks.

By the death of Miss Emily Angove, Matron of the West Cornwall Miners' Hospital and Women's Hospital, at Redruth, this post is rendered vacant. At the annual meeting of the subscribers to the Women's Hospital, presided over by Mrs. Basset, of Tehidy, a resolution of sympathy with the relatives of Miss Angove, and regret at her death was recorded in the minutes. The Rev. H. Oxland, who seconded the motion, spoke of the regret which the working men who had been under the late Matron's care felt at her decease. The men spoke not only of her great kindness as an official in the Miners' Hospital, but of her real personal goodness. At the annual meeting of the subscribers to the West Cornwall Miners' Hospital, the following week, the Committee, in their annual report, "bore testimony to the cleanliness and order in which the late Matron (Miss Angove) kept the hospital, and the interest she always took in the patients under her charge, by all of whom she would be mourned as a sincere friend, who spent all her energies in caring for them while there, and did not forget them when they returned to their homes and occupations." It was resolved that a copy of this portion of the report be forwarded to the late Matron's relatives.

Many who have worked with and under Sister Annie de Terraneau will learn with great regret of her death, after only a few days' serious illness, at Thorpe Hall, Winston, Yorkshire, on March 1st. She was trained at the Royal Southern Hospital, Liverpool, in 1889, where she afterwards held the post of Night Sister for fourteen months. Subsequently she worked at the Macclesfield Infirmary, and at the Newcastle District Nurses' Home, but owing to ill-health she was obliged at the close of the year to abandon work for the time. In her the nursing profession has lost one who combined a keen sense of duty with unwearying endeavour.

### Appointments.

Colonial Hospital, Gibraltar.—Miss J. Stirling Hamilton, late night Sister London Hospital, to be Lady Superintendent. Miss Emma Morley (late night Nurse Queen) to be night Sister. Miss Agnes Kelly (late Nurse Sophia), Miss Hariette Towell (late Nurse Redman), Miss Caroline Peacock (late Nurse Queen) to be ward Sisters. All trained at the London Hospital, and sailed Friday, March 11th.

Colonial Hospital, Hong Kong.—Miss Gertrude Brooke, trained at the London Hospital, has been appointed Sister at Hong Kong, and sailed on March 5th.

Eastern Hospitals, Homerton.—Miss Kate Mackenzie has been appointed Matron of this hospital. Miss Mackenzie trained at St. Marylebone Infirmary, Notting Hill, passing through all the grades of probationer, nurse, and Sister; she subsequently served two years as Assistant Matron at the Brownlow Hill Infirmary, Liverpool.

The Peake Hospital, Hong Kong.—Miss Mary Agnes Thompson has been appointed Matron of this hospital. Miss Thompson also trained at St. Marylebone Infirmary, where she remained several years, rising from probationer to nurse and Sister; she left eighteen months ago, to take the post of nursing Sister at the Civil Hospital, Hong Kong, which post she resigns on her present appointment.

Plymouth Cottage Hospital.—Miss Layon (Sister Frances), of the London Homœopathic Hospital, has been appointed Matron-Nurse to this little institution in Flora Place.

Radstock.—Miss C. Anderson has been appointed Sick Visiting Nurse to the town of Radstock. Miss Anderson was trained in the Royal Infirmary, Edinburgh, and has also been for some years at the "Sarah Acland Home," Oxford.

Royal Naval Hospital, Stonehouse.—Nursing Sister Grace Mackay, late of Malta, has been appointed in charge of the above hospital.

### Off Duty

### Ugliness versus Beauty.

Ugliness? Well, that is, perhaps, rather a hard word to use—at least of a woman. Man has a prescriptive right to be ugly, if only he be "interesting"; but a woman whose face is the reverse of beautiful will usually escape with the epithet "unfortunately plain." Whether she really benefits by this may be a question, for there is often a certain piquancy in ugliness, while the word "plain" seems to suggest hopeless commonplaceness. Still, this euphemism is meant politely; and in these days, when men—young men, at any rate—have far less chivalrous respect in their manner towards women than in the good old days, one must be thankful for small mercies. Besides, the question before us is one, not of words, but of things. Ugliness, or Plainness (which you will), comes into court to plead against Beauty; let us hear what she has to say for herself.

It is a bold proceeding, for the world is on Beauty's side—there is no doubt of that. Charlotte Brontë made a gallant, and to some extent successful, stand against the prevailing opinion when she introduced her "Jane Eyre" into society, but we have fallen back again since then; and now, if our heroines are not beautiful to start with—a concession which the author has evidently much difficulty in bracing himself up to—they always become so when they reach full womanhood, or else when they are pierced by Cupid's darts. There is no need, then, to hear what Beauty has to say for herself; we all think well, perhaps too well, of her; we all know what a power she is—how she softens the hard heart, how she draws all men to her feet, and bids them pour out their treasures before her,—wealth, fame, and honour, sometimes even life itself. Let us turn to Ugliness then, and see whether she can make out a claim to a portion of our affection and regard. A small portion, indeed, is all she asks; but at present, she declares, Beauty has usurped it all.

First of all, Ugliness urges that she is a valuable preservative against self-deception and disappointment. Her possessors stir no surface feeling, and therefore, if they stir any feeling at all, they may well hope that it is deep and lasting. "I do pity the poor thing," was said once of a married woman who had no good looks to boast of. "Her husband seems devoted to her now; but I can't help being afraid that some day he will wake up and see how dreadfully ugly she is, and then love her no longer." Now, surely, this pity was entirely misplaced. If a woman is ugly, and *yet* wins for herself affection, she is in far less danger of losing that affection than is many a beautiful woman. The fact that, in spite of her ugliness, she proved attractive, shows that she has something in her nature capable of winning real love; and the beautiful woman may be entirely without that something—how can she, indeed, be quite sure that she possesses it, so long as her beauty lasts? There is something truly tragic about the fate of a girl whose beauty so attracts a man that he declares (and feels) that without her love the world would be a wilderness to him, and who, when she has once accepted him, finds the glamour of her beauty wearing off, and herself powerless to interest him. Who is to blame? She for giving her love to so ardent a wooer, or he for failing to discover that she had nothing in her which could permanently satisfy his mind and heart? "Neither, neither!" says Ugliness; "it was Beauty who was to blame."

And certainly, if we admit this, Beauty has to answer for many a wretched life, many a broken heart; for these poor victims of hers, though they may be mindless, and wanting in force of character, have very often tender and sensitive hearts, and can suffer with the best.

Again, says Ugliness, Beauty must pass away. Her spells may sometimes be so potent that want of mind, want of heart, want of everything but this one charm, may be overlooked; but any day she may fade away; one day she must. What then? Can you live upon the remembrance of what has been? That is but a poor prospect, and yet it is all that some beautiful women can hope for, while none can be absolutely certain, so long as their beauty lasts (and this is where, to our thinking, Ugliness makes her point), how much will be left to them when at length she takes to herself wings and flies away.

Ugliness has no such fears. "As the past has been, so will the future be," she says to herself. "What I have borne, I can bear; what I have gained, I may hope to keep." True, she admits, and admits with sorrow, that she may prevent some of her possessors from ever having full scope for the rare powers of heart and mind which lie concealed beneath that unattractive exterior. Still, she contends that beauty is by no means so all-important as some people would make out. On children, at any rate, external appearance seems to have but little effect. Nothing is prettier than to see a little child mothering some hideous doll, or clinging to, and covering with kisses, a nurse whose hard unattractive features you would have thought enough to repel any such advances. Love, and the "sweet habit of kindness" have so triumphed over Ugliness, that it is as though she were not; and so it will still be in many and many a case, not only with children, but with men and women as well. Who cannot recall an instance of a plain or ugly nurse who is adored by her patients and family, of whom it might be said with truth, "Beauty she has none, and yet to many hers is the loveliest face in the world"?

Lastly, Ugliness claims that she plays no insignificant part in developing beauty of character, in producing humility, gentleness, and unselfishness. A beautiful woman is often apt to think that she need not trouble herself to talk or make herself pleasant,—that she has nothing to do but to stand and receive her meed of admiration; while, if she happens not to receive it, she feels injured and resentful. An ugly woman, on the other hand, does not think of herself, or if she does, it is only to notice—as she cannot help doing sometimes—how little account society makes of her, or to be grateful for some small attention which has been paid her. It is she, very often, who keeps things going; she who sees to guests who seem to be rather left out in the cold; she who, having "a heart at leisure from itself," is called upon to sympathise with the joys and sorrows of others. Thus is generated a real loveliness of character which, to the seeing eye and understanding heart, far surpasses outward beauty.

That there are not wanting such eyes, even among poets—with whom outward beauty, one might think, is almost a *sine quâ non*—the following touching little sonnet, by Cosmo Monkhouse, shows:—

On One Not Beautiful.

"Dear Soul, how different were you from those
Who, clothed in more than mortal loveliness,
Have but to speak, or move, or smile, t'express
The virtue rare their eloquent forms enclose!
More different still from them whose beauty throws
A glamour round their real unsightliness,
With hearts less tender than their least caress,
And minds less graceful than their idlest pose.
O Soul most beautiful! to whom was given
A form that hid you as a cloud a star,
Bearing no semblance to the light disguised,
When you within the crystal streams of Heaven
Shall see yourself as lovely as you are,
How happy you will be, and how surprised!"

## Figures and Tables

**Figure f1:**
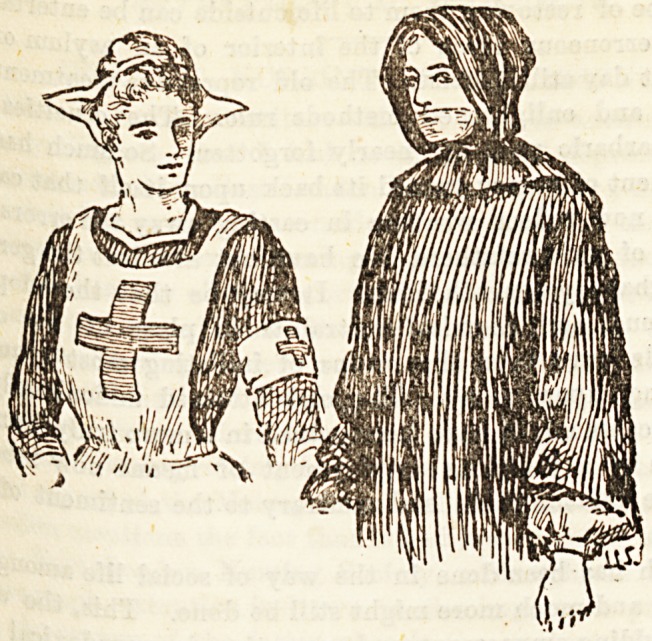


**Figure f2:**